# Delegates to the American Medical Association

**Published:** 1850-02

**Authors:** 


And the following were elected Delegates to the American Medical Association:

Caspar Morris, R. La Roche, Charles D. Meigs, Wm. H. Klapp, W. S. W. Ruschenberger, John Rodman Paul, Charles Evans, B. H. Coates, Wm. Byrd Page, Francis Gurney Smith, Lewis Rodman, Samuel L. Hollingsworth.

